# Trachymyrmex septentrionalis Ant Microbiome Assembly Is Unique to Individual Colonies and Castes

**DOI:** 10.1128/msphere.00989-21

**Published:** 2022-07-07

**Authors:** Emily A. Green, Jonathan L. Klassen

**Affiliations:** a Department of Molecular and Cell Biology, University of Connecticut, Storrs, Connecticut, USA; b Institute for Systems Genomics, University of Connecticut, Storrs, Connecticut, USA; University of Michigan-Ann Arbor

**Keywords:** fungus-growing ants, microbial ecology, microbiome, mollicutes, social insects

## Abstract

Within social insect colonies, microbiomes often differ between castes due to their different functional roles and between colony locations. Trachymyrmex septentrionalis fungus-growing ants form colonies throughout the eastern United States and northern Mexico that include workers, female and male alates (unmated reproductive castes), larvae, and pupae. How *T. septentrionalis* microbiomes vary across this geographic range and between castes is unknown. Our sampling of individual ants from colonies across the eastern United States revealed a conserved *T. septentrionalis* worker ant microbiome and revealed that worker ant microbiomes are more conserved within colonies than between them. A deeper sampling of individual ants from two colonies that included all available castes (pupae, larvae, workers, and female and male alates), from both before and after adaptation to controlled laboratory conditions, revealed that ant microbiomes from each colony, caste, and rearing condition were typically conserved within but not between each sampling category. Tenericute bacterial symbionts were especially abundant in these ant microbiomes and varied widely in abundance between sampling categories. This study demonstrates how individual insect colonies primarily drive the composition of their microbiomes and shows that these microbiomes are further modified by developmental differences between insect castes and the different environmental conditions experienced by each colony.

**IMPORTANCE** This study investigates microbiome assembly in the fungus-growing ant *Trachymyrmex septentrionalis*, showing how colony, caste, and lab adaptation influence the microbiome and revealing unique patterns of mollicute symbiont abundance. We find that ant microbiomes differ strongly between colonies but less so within colonies. Microbiomes of different castes and following lab adaptation also differ in a colony-specific manner. This study advances our understanding of the nature of individuality in social insect microbiomes and cautions against the common practice of only sampling a limited number of populations to understand microbiome diversity and function.

## INTRODUCTION

Social insects live in colonies and manifest group integration, division of labor, and overlap of generations (1). These divisions of labor are often distributed between developmental castes, which at the most basic level are divided into reproductive individuals and sterile workers, which themselves can be further partitioned into specialized groups for different tasks. Gut microbiomes often differ between social insect castes, possibly due to the specialized functional role played by each caste (2). For example, the microbiomes of termite minor workers, major workers, and soldiers all differ from each other (3, 4), and termite reproductive castes have unique microbiomes compared to those of workers from the same colony (5–8). Honey bee queens, workers, and drones also each have unique gut microbiomes, where worker microbiomes are more diverse than those of queens and drones, possibly due to worker foraging (9). Honey bees have a well-defined core microbiome that is found in all colonies and castes, including queens, workers, and drones (10, 11). However, strains varied between geographic locations, individual colonies, and bee castes (9, 11). Social insect microbiome composition is therefore determined by a complex combination of developmental differences and colony-specific ecological factors.

Fungus-growing ants are social insects (Formicidae: tribe Attini) that evolved 55 to 65 million years ago (MYA) in South America (12–14). Today there are 19 genera, including approximately 250 known species that range throughout North and South America from the northern United States to southern Argentina (14–18). The ~250 species of fungus-growing ants are separated into five separate agricultural systems representing transitions in their evolution: lower, coral-fungus, yeast, higher, and leaf-cutter agriculture (14). Fungus-growing ant caste systems include workers, reproductive queens, and unmated reproductive alates. Leaf-cutting ants (*Atta*, *Acromyrmex*, and *Amiomyrmex*) can have multiple worker castes, including soldier and major, media, and minor workers (16). All fungus-growing ants grow a cultivar fungus, typically a species of *Leucoagaricus* (except for in coral-fungus agriculture by *Apterostigma* ants, which cultivate a Pterulaceae species [19]). This cultivar serves as the ants’ main food source and has coevolved with the ants for millions of years (12, 13, 15, 16). Ants forage for materials to feed to their cultivar fungus (e.g., grass, leaves, dried plant material, and/or insect frass), which the fungus degrades and converts to fungal biomass, including hyphal swellings called gonglydia that the ants eat as their primary food source (20, 21). *Pseudonocardia*, an actinobacterium, grows on the cuticle of many fungus-growing ants, producing antimicrobials to protect the fungus garden from pathogens (22, 23). Other cuticular microbes on the ants have been identified, but their function is unknown (23, 24). Together these interactions make up a multipartite symbiosis composed of the fungus-growing ant, the *Leucoagaricus* fungus, and the protective symbiont *Pseudonocardia.*

Several studies have preliminarily investigated the fungus-growing ant microbiome, primarily focusing on leaf-cutting ants (25–30). These studies show that *Acromyrmex* and *Atta* leaf-cutting ants host low-diversity microbiomes that include *Wolbachia*, *Solirubrobacter*, Enterobacter, Pseudomonas, and members of the orders *Entomoplasmatales* and *Rhizobiales*. *Rhizobiales* bacteria contain *nif* genes that were hypothesized to cycle nitrogen for the ants and their cultivar fungus (29), as do *Methylobacterium*, *Ralstonia*, and Pseudomonas strains (30). *Wolbachia* is maternally transmitted in *Acromyrmex* and *Atta* ants but lacks a known function. *Entomoplasmatales* bacteria belong to the genera *Mesoplasma* and *Spiroplasma* and are thought to provide nutrients to the ants (28, 31). Most of these studies pooled multiple worker ants together before DNA extraction, leaving how ant microbiomes differ between individuals within a colony and between castes largely unknown.

Here, we characterize the microbiome of the fungus-growing ant Trachymyrmex septentrionalis. Phylogenetically, the higher agriculture ant genus *Trachymyrmex* holds a transitional position adjacent to leaf-cutting ants (17). Typical *T. septentrionalis* colonies have approximately 1,000 workers in addition to a single queen, and during the summer months they also produce reproductive female and male alates that remain in the colony before mating as a mass swarm (16). *T. septentrionalis* colonies are found in pine barren and sandy soil ecosystems throughout the eastern United States and northern Mexico (20, 32, 33) and vary genetically across the United States, forming four different clades (33). Ishak et al. (34) sampled *T. septentrionalis* ants from 25 colonies at a single location in Texas over 1 year and identified 19 bacterial genera that were conserved in *T. septentrionalis* workers and alates (both males and females). They also identified differences in ant microbiomes between castes, albeit including only a few samples. However, the samples collected from each colony were not differentiated from each other, making it impossible to study intracolony variation in microbiome composition. Whether the 19 common bacteria found in Texas *T. septentrionalis* form a conserved microbiome that is found in other geographic regions or castes is also unknown.

In contrast to this previous work, we sampled individual ants collected across a broad geographic gradient to characterize the conserved microbiome of field-collected *T. septentrionalis* worker ants. We also more deeply sampled individual ant microbiomes within two colonies and found that ant gut microbiome composition differs between colonies, between ant castes, and following adaptation of colonies to the laboratory environment. The variable presence of *Mesoplasma* and *Spiroplasma* symbionts in different ant colonies and castes was a major driver of these microbiome differences. Our study provides an example of how individual colonies, castes, and ecological differences determine the composition of social insect microbiomes.

## RESULTS

### Ant microbiomes differ between states.

**FIG 1:**
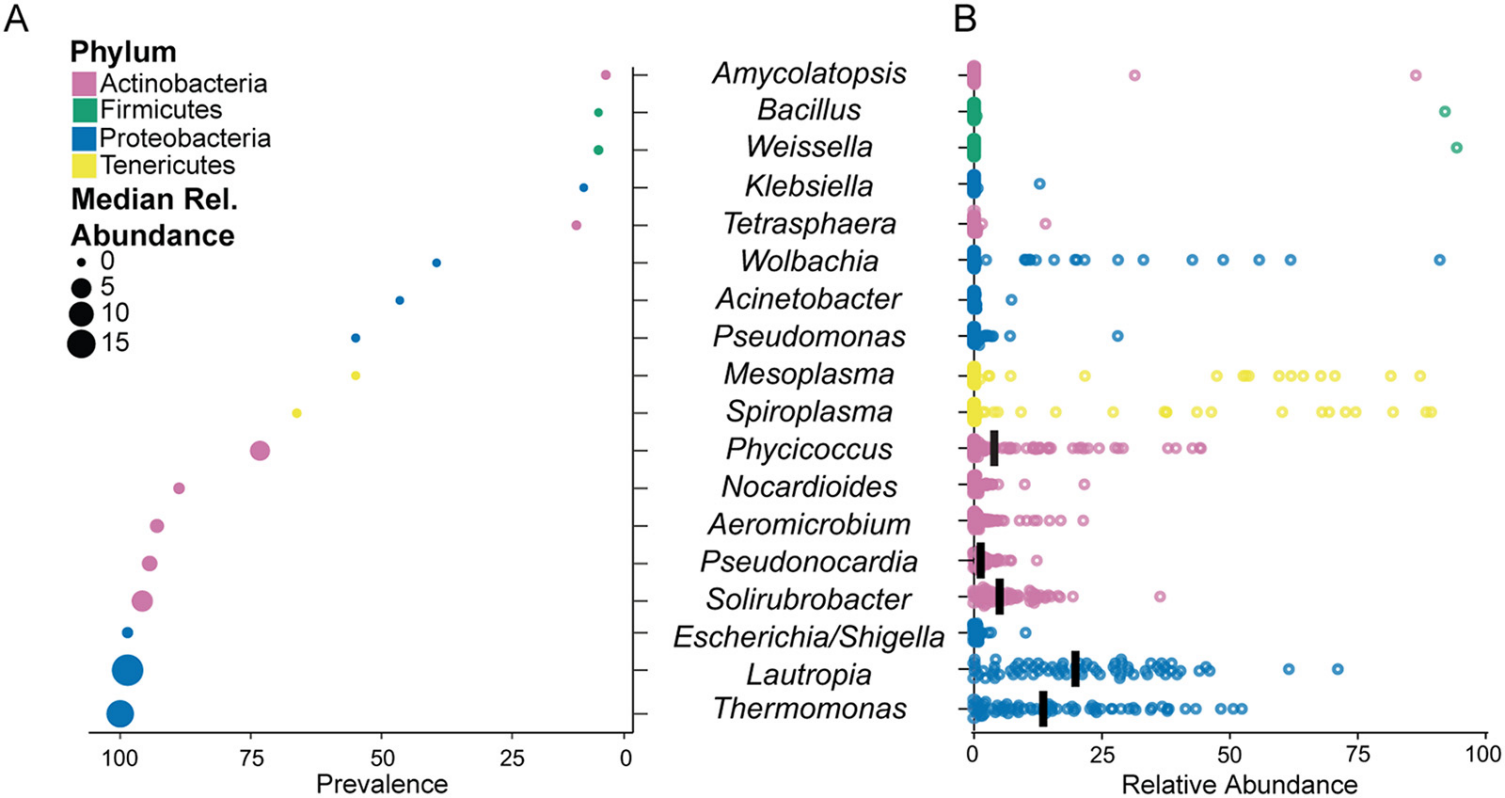
The *T. septentrionalis* worker ant conserved microbiome. (A) Prevalence of bacterial genera in the multistate data set with an abundance of ≥5% in at least one ant microbiome. Genera are ordered on the *y* axis from most to least prevalent. Median relative abundance is represented by circle size, and phylum is represented by color. (B) Relative abundance of the bacterial genera of ≥5% in at least one ant sample in the multistate data set, with the abundance in each sample represented by a single point for each genus. Bars indicate medians.

We characterized *T. septentrionalis* worker ant microbiomes in field-collected ants from New York, down the U.S. East Coast into Florida, and westward into Louisiana (see Fig. S1 in the supplemental material, hereafter referred to as the multistate dataset). *T*. *septentrionalis* worker ant microbiomes were dominated by bacteria belonging to the phyla *Actinobacteria*, *Firmicutes*, *Proteobacteria*, and *Tenericutes* (Fig. 1A; Fig. S3). Alpha diversity was low for these *T. septentrionalis* microbiomes and did not differ between states or between ants sampled from the same colony (Fig. S4A and B; analysis of variance [ANOVA]: state *P* = 0.492, colony *P* = 0.335). A total of 18 genera were present with an abundance of ≥5% in at least one sample (Fig. 1A and B). The most prevalent genera in the data set, making up the conserved *T. septentrionalis* worker ant microbiome, were *Thermomonas*, *Lautropia*, Escherichia, *Solirubrobacter*, *Pseudonocardia*, *Aeromicrobium*, and *Nocardioides* (Fig. 1A). These bacteria had a prevalence of at least 75% throughout the data set and cooccurred frequently. *Phycicoccus*, *Mesoplasma*, *Spiroplasma*, Acinetobacter, and *Wolbachia* were less prevalent in this data set (50 to 75%; Fig. 1A). The relative abundances of all genera varied greatly between samples (Fig. 1B). *Pseudonocardia*, *Aeromicrobium*, *Nocardioides*, and *Solirubrobacter* each had high prevalences but low median relative abundances (<25%) in most samples (Fig. 1B). *Thermomonas* and *Lautropia* had high median relative abundances that varied greatly between samples. When present, *Mesoplasma* and *Spiroplasma* were often highly abundant (>50% of all microbes in a sample) and did not cooccur with one another in any sample. Many ant colonies sampled from New York and North Carolina lacked *Mesoplasma* or *Spiroplasma* (Fig. S3). *Amycolatopsis*, *Bacillus*, and *Weissella* were also highly abundant in 1 to 2 samples (Fig. 1B).

10.1128/msphere.00989-21.1 FIG S1 Maps detailing colony locations. The left map shows the location of the colonies in the multistate data set and two-colony data set using black circles. On the right, New Jersey is magnified to show colonies from the multistate data set (black circles) and the two-colony data set (green circles). Maps were created using www.simplemappr.net/ and edited in Adobe Illustrator. Download FIG S1, TIF file, 1.2 MB. Copyright © 2022 Green and Klassen. 2022 Green and Klassen. https://creativecommons.org/licenses/by/4.0/ This content is distributed under the terms of the Creative Commons Attribution 4.0 International license.

10.1128/msphere.00989-21.3 FIG S3 Bar plot of the phyla found in samples from the multistate dataset. Single bars represent a single ant microbiome and are grouped by state of collection. The *x* axis brackets group worker ants from the same colony. Phyla are differentiated by color, and “other” represents phyla present at >15% relative abundance. *n* = 71. Download FIG S3, TIF file, 1.0 MB. Copyright © 2022 Green and Klassen. 2022 Green and Klassen. https://creativecommons.org/licenses/by/4.0/ This content is distributed under the terms of the Creative Commons Attribution 4.0 International license.

10.1128/msphere.00989-21.4 FIG S4 (A) Shannon and Simpson alpha diversities for samples in the multistate data set. Median alpha diversities are marked with diamonds. (B) ANOVA of alpha diversity scores for the multistate data set, compared between states and colonies. *n* = 71. Download FIG S4, TIF file, 1.2 MB. Copyright © 2022 Green and Klassen. 2022 Green and Klassen. https://creativecommons.org/licenses/by/4.0/ This content is distributed under the terms of the Creative Commons Attribution 4.0 International license.

**FIG 2:**
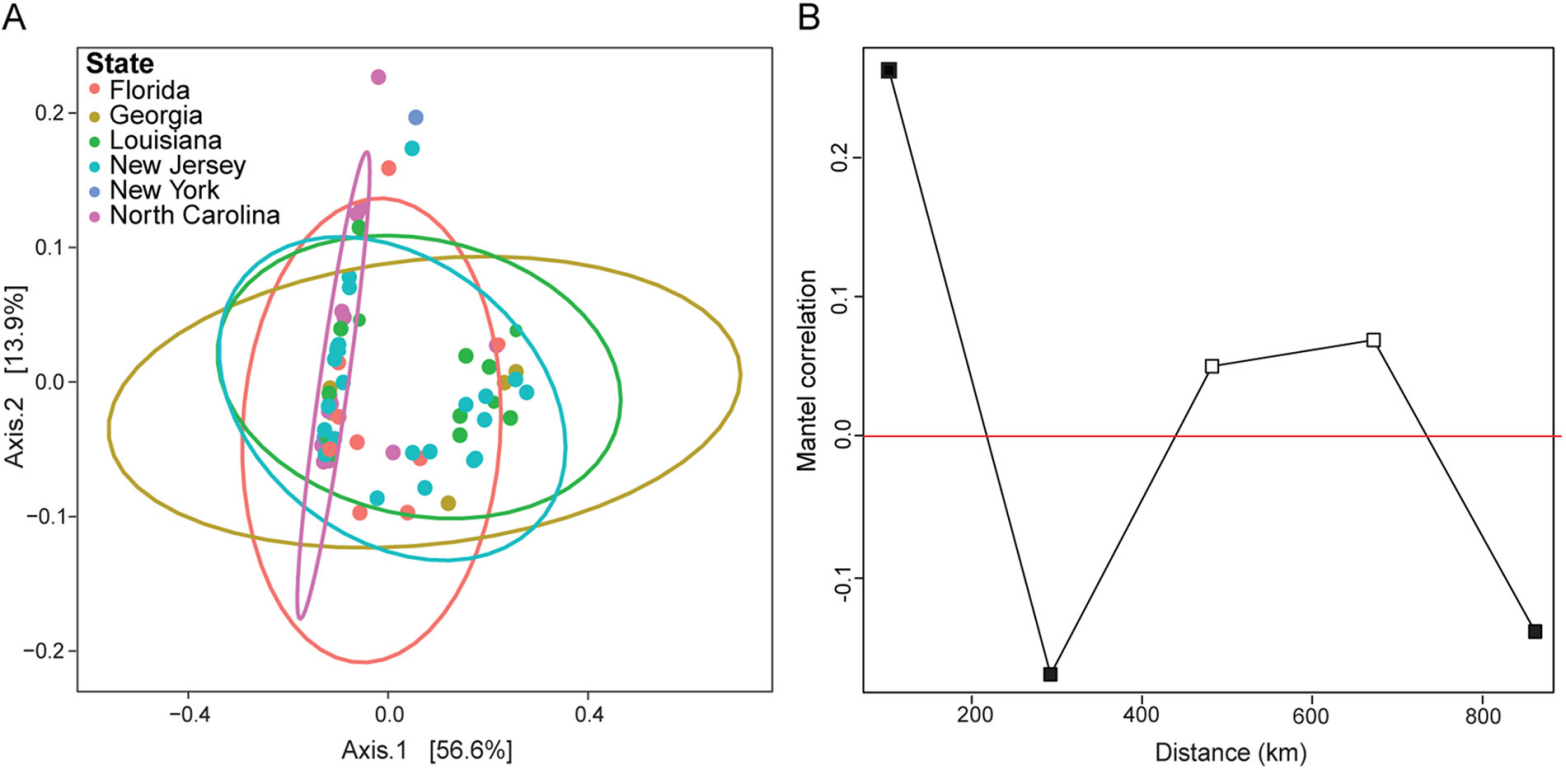
Biogeography of the *T. septentrionalis* worker microbiome. (A) PCoA of weighted Unifrac distances between ant microbiomes in the multistate data set. Samples are colored by state and grouped using a multivariate *t*-distribution. *n* = 71. (B) Partial Mantel correlation between unweighted Unifrac distances and ant sampling locations in the multistate data set, excluding comparisons between ants sampled from the same colony. The *x* axis indicates the distance class index in kilometers, and the *y* axis indicates the Mantel correlation R statistic. Filled and unfilled squares indicate significant (*P* < 0.05) and nonsignificant (*P* > 0.05) *P* values, respectively, and points above and below the red line indicate positive and negative correlations, respectively.

Multiple ants were collected from most colonies in the multistate data set, and the colony from which ants were collected explained more variation in microbiome composition than did the state of collection (weighted Unifrac [WUF] colony permutational multivariate analysis of variance [PERMANOVA]: R^2^ = 0.524, *P* = 0.003; WUF state PERMANOVA: R^2^ = 0.135, *P* = 0.003; Fig. S5B). These trends remained after excluding samples from New York and North Carolina, which lacked *Tenericutes* (Fig. S5B). Although ant microbiomes from the multistate data set clustered weakly by state (Fig. 2; Fig. S5A), WUF distances between ant microbiomes did not correlate with the geographic distances between collection locations, although unweighted Unifrac (UUF) distances did, albeit weakly (UUF Mantel *R* = 0.143, *P* = 0.019, Fig. 2B; WUF Mantel *R* = 0.034, *P* = 0.778, Fig. S5C and D). In the corresponding partial Mantel tests, distances between colonies from the smallest distance class (<100 km) were positively and significantly correlated with UUF distances between the ant microbiomes from these colonies (Fig. 2B; Fig. S5D). Distance from classes 2 (~250 km) and 5 (~850 km) negatively and significantly correlated with UUF distances in ant microbiome composition, but all other correlations were nonsignificant (Fig. 2B; Fig. S5C and D). Overall, these analyses show that microbiomes from colonies collected within 100 km from each other were somewhat similar to each other but that microbiomes otherwise varied in composition between colonies.

10.1128/msphere.00989-21.5 FIG S5 (A) PCoA of unweighted Unifrac distances between ant microbiomes in the multistate data set. Samples are colored by state and grouped using a multivariate *t*-distribution. (B) PERMANOVA analyses testing the variation of ant microbiomes in the multistate data set explained by state of collection (New York, New Jersey, North Carolina, Georgia, Florida, and Louisiana) or colony of origin. The bottom statistics exclude North Carolina and New Jersey, which lacked *Tenericutes* symbionts. (C) Mantel correlation plot of weighted Unifrac distances and ant colony geographic location (GPS points) of the multistate data set. The *x* axis is the distance class index in kilometers, and the *y* axis is the Mantel correlation R statistic. Filled squares indicate significant *P* values (*P* < 0.05), and unfilled squares indicate nonsignificant *P* values (*P* > 0.05). Points above and below the red line indicate positive and negative correlations, respectively. (D) Partial Mantel statistic scores for the comparison of microbiome similarity and geographic distance in the multistate dataset. For panels B and C), significance codes: ***, *P* ≥ 0.001; **, *P* ≥ 0.01; *, *P* ≥ 0.05.s. Download FIG S5, TIF file, 1.8 MB. Copyright © 2022 Green and Klassen. 2022 Green and Klassen. https://creativecommons.org/licenses/by/4.0/ This content is distributed under the terms of the Creative Commons Attribution 4.0 International license.

### Ant microbiomes differ between castes and due to lab adaptations.

**FIG 3:**
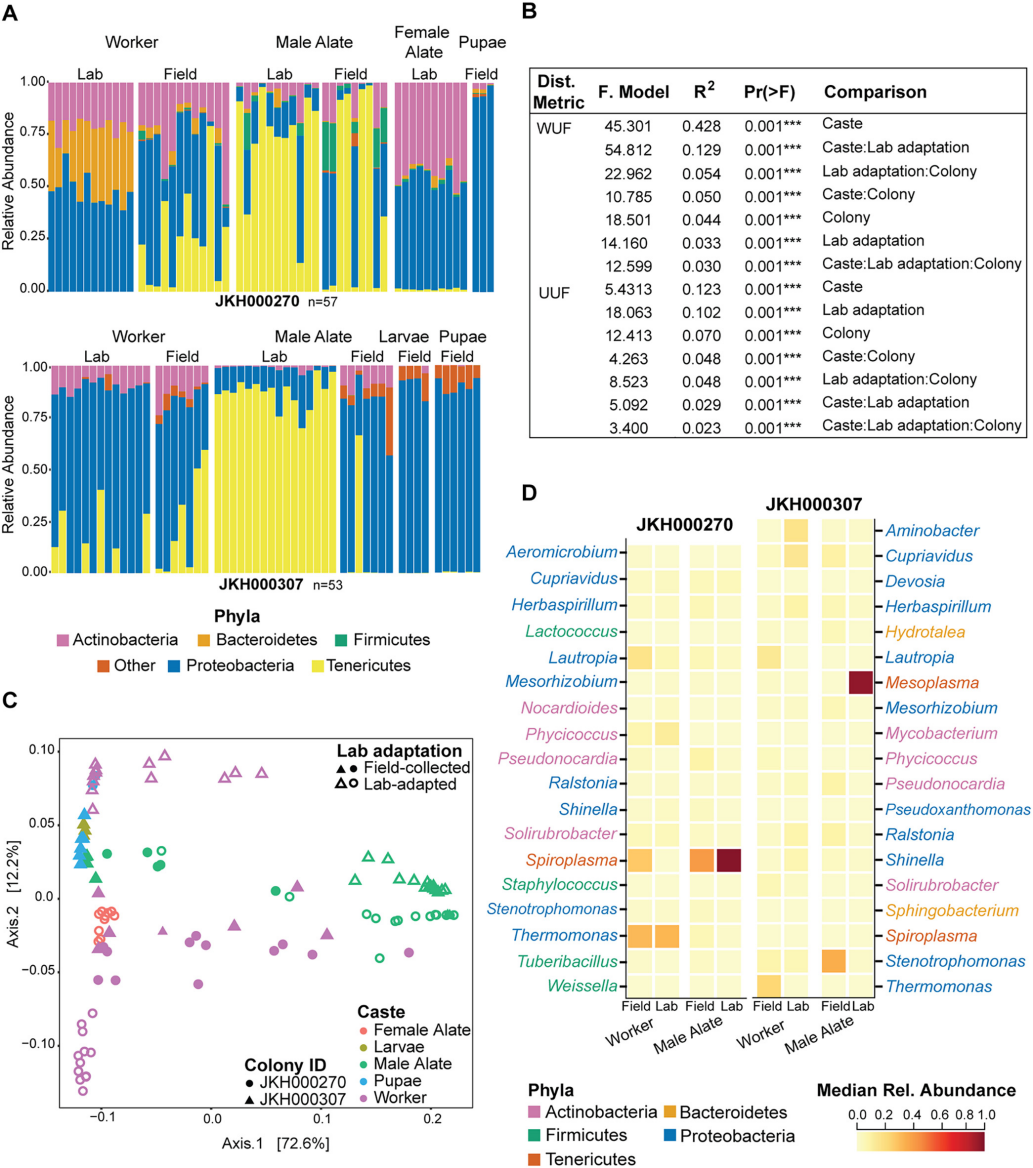
Colony, caste, and lab adaptation all determine ant microbiome composition. (A) Bar plot of the most abundant phyla in microbiomes from colonies JKH000270 (top) and JKH000307 (bottom). Single bars represent individual ant microbiomes, and sample types are grouped along the *x* axis. The *y* axis indicates relative abundance. Phyla are differentiated by color, and “other” represents phyla present at <15% relative abundance. (B) PERMANOVA analyses testing correlations between ant microbiome composition in the two-colony data set and ant caste, lab adaptation status, and their colony of origin. *n* = 110. (C) PCoA of weighted Unifrac distances between ant microbiomes in the two-colony data set. Colors indicate ant caste, shapes indicate colony ID, and solid and open shapes indicate field-collected and lab-maintained samples, respectively. (D) Heatmap of genera with an abundance of ≥5% in worker ants and male alates from colony JKH000270 (left) and colony JKH000307 (right). Genera are listed on the *y* axis, and colors indicate their phylum.

In our multistate data set, we found that ant microbiomes were more conserved within a colony than between colonies. However, the multistate data set only included 1 to 3 field-collected worker ants to represent each colony. To overcome this limitation, we more deeply sampled individual ants from two colonies collected in Wharton State Forest, New Jersey (supplemental File 1, available at https://github.com/klassen-lab/Green_2022). The field-collected workers in this two-colony data set had low alpha diversities, similar to those in the multistate data set (Fig. S6A). We also found that the same phyla were conserved in the two-colony data set as in the multistate data set. Field-collected worker ants were colonized by *Proteobacteria*, *Tenericutes*, and *Actinobacteria*, with some ants from colony JKH000270 also colonized with *Bacteroidetes* (Fig. 3A). Most conserved worker ant microbiome genera from the multistate data set (*Thermomonas*, *Lautropia*, *Solirubrobacter*, *Pseudonocardia*, *Aeromicrobium*, and *Nocardioides*) were also present in field-collected worker ants in the two-colony data set (Fig. S7).

10.1128/msphere.00989-21.6 FIG S6 (A) Shannon and Simpson alpha diversities for colonies JKH000270 and JKH000307. The *x* axes list each caste, and median alpha diversities are marked with diamonds. (B) ANOVA of alpha diversity scores for the two-colony data set, compared between colonies JKH000270 and JKH000307, caste, and whether ants were lab-maintained or sampled in the field. Significance codes: ***, *P* ≥ 0.001; **, *P* ≥ 0.01; *, *P* ≥ 0.05. *n* = 110. Download FIG S6, TIF file, 2.9 MB. Copyright © 2022 Green and Klassen. 2022 Green and Klassen. https://creativecommons.org/licenses/by/4.0/ This content is distributed under the terms of the Creative Commons Attribution 4.0 International license.

10.1128/msphere.00989-21.7 FIG S7 Heatmap of bacterial genera in the two-colony data set with an abundance of ≥5% in at least one sample. On the left, a Euclidian distance dendrogram represents the relationship between the relative abundance of reads in each genus, labeled on the right. The top dendrogram clusters the ant microbiomes using the ward.D distance. On the bottom, colors in the three different rows differentiate samples by their colony, caste, and whether they are lab-adapted. *n* = 110. Download FIG S7, TIF file, 2.0 MB. Copyright © 2022 Green and Klassen. 2022 Green and Klassen. https://creativecommons.org/licenses/by/4.0/ This content is distributed under the terms of the Creative Commons Attribution 4.0 International license.

Although the limited number of colonies in the two-colony data set is not ideally powered to compare ant microbiomes from different colonies in a general way (in contrast to the multistate data set), it instead prioritizes sampling of ants from different developmental stages and castes (pupae, larvae, male alates, and female alates) and ants collected before and after lab adaptation (field-collected or lab-maintained). The alpha diversity of lab-maintained male alates was often significantly lower than that of other sample types, which otherwise differed minimally from each other (Fig. S6B). Like the workers, all castes were colonized by *Actinobacteria*, *Proteobacteria*, and *Tenericutes*, with *Bacteroidetes* only abundant in colony JKH000270, along with *Firmicutes* in some male alates (Fig. 3A). Compared to the worker microbiomes, *Tenericutes* were particularly abundant in male alate microbiomes, *Proteobacteria* in pupae and larvae, and *Actinobacteria* and *Proteobacteria* in female alates (Fig. 3A). There were extremely few *Mesoplasma* or *Spiroplasma* organisms in pupae and larvae from the two-colony data set. However, *Mesoplasma* and *Spiroplasma* were abundant in the separate pupae data set generated from other *T. septentrionalis* colonies and used for confirmation (Fig. S8A).

10.1128/msphere.00989-21.8 FIG S8 (A) Bar plot of the top phyla in pupae from colony JKH000125 (left) and JKH000136 (right). Phyla are differentiated by color, and “other” represents phyla present at <15% relative abundance. (B) *Spiroplasma* and *Mesoplasma* relative abundances in pupae from colonies JKH000136 and JKH000125. (C) *Mesoplasma* and *Spiroplasma* relative abundances in pupae from colonies JKH000125 and JKH000136. Single bars represent abundances in single pupae, and the *x* axis brackets group pupae from each colony. Samples shown to the right of the figure were ethanol washed before DNA extraction, unlike those on the left. Download FIG S8, TIF file, 1.6 MB. Copyright © 2022 Green and Klassen. 2022 Green and Klassen. https://creativecommons.org/licenses/by/4.0/ This content is distributed under the terms of the Creative Commons Attribution 4.0 International license.

In the two-colony data set, ant microbiome composition differed between all tested categories, and although caste explains the most variation, it depends on the colony and lab adaptation (Fig. 3B and C). Caste accounted for the most microbiome variation using both WUF and UUF distances (Fig. 3B). These differences were colony-, caste-, and adaptation-specific because all interaction terms accounted for substantial microbiome variation using both WUF and UUF distances. Microbiomes from colonies JKH000270 and JKH000307 clustered separately along axis 2 in the WUF and UUF principal-coordinate analyses (PCoAs) but overlapped in the center (Fig. 3C; Fig. S9). This second axis accounts for a smaller amount of microbiome variation than axis 1, which separates the microbiomes into different castes, lab adaptation statuses and combinations of these categories in both the WUF and UUF PCoAs.

10.1128/msphere.00989-21.9 FIG S9 PCoA of unweighted Unifrac distances between ant microbiomes in the two-colony data set. Colors indicate ant caste, shape indicates colony ID, and solid and open shapes indicate field-collected and lab-maintained samples, respectively. *n* = 110. Download FIG S9, TIF file, 0.7 MB. Copyright © 2022 Green and Klassen. 2022 Green and Klassen. https://creativecommons.org/licenses/by/4.0/ This content is distributed under the terms of the Creative Commons Attribution 4.0 International license.

Although each caste had a unique microbiome, we could only directly compare changes resulting from lab adaptation for workers and male alates, which were the only castes that we sampled from both field and lab settings (Fig. 3D). In colony JKH000270, field-collected and lab-maintained worker and male alate microbiomes differed principally in their abundances of *Spiroplasma* (Fig. 3D). In colony JKH000307, field-collected and lab-maintained worker microbiomes differed in their abundances of *Thermomonas*, *Lautropia*, *Aminobacter*, and *Cupriavidus*, and lab-maintained, but not field-collected, male alate microbiomes were dominated by *Mesoplasma* (Fig. 3D). Lab-maintained microbiomes were much less variable than the field-collected microbiomes, except for colony JKH000307 worker ants, when measured using UUF distances (Fig. S10A and B).

10.1128/msphere.00989-21.10 FIG S10 (A) Box and whisker plots comparing distributions of weighted (right) and unweighted (left) Unifrac distances for lab-maintained and field-collected samples from each caste and colony. Boxes represent the upper and lower quartiles, whiskers represent the maximum and minimum values, and the middle bar represents the median. Brackets between two samples indicate a difference of statistical significance. Significance codes: ***, *P* ≥ 0.001. (B) Analyses testing if the variation in weighted or unweighted Unifrac distances differ between lab-maintained and field-collected microbiomes, comparing only castes and colonies for which both lab-maintained and field-collected samples were available. (C) Median weighted (top) and unweighted (bottom) Unifrac distances within and between samples from the same caste and lab-adaptation categories for each colony. Colors indicate median Unifrac distances between samples belonging to each category. Download FIG S10, TIF file, 2.9 MB. Copyright © 2022 Green and Klassen. 2022 Green and Klassen. https://creativecommons.org/licenses/by/4.0/ This content is distributed under the terms of the Creative Commons Attribution 4.0 International license.

In nearly all instances, microbiomes varied more between each category (colony, caste, and lab adaption) than within the same category (Fig. S10C). Microbiomes from each colony were grouped by caste, and then worker and male alate samples were further grouped into lab-maintained and field-collected categories to remove lab adaptation as a potentially confounding variable (Fig. S10C). Typically, median WUF and UUF distances were greater between categories (caste, colony, lab adaptation) than within them. However, this was not true for UUF JKH000270 field-collected male alates, JKH000270 female alates, and JKH000307 larvae (Fig. S10C). Overall, these comparisons highlight that ant microbiomes are more similar within our sampling categories (colony, caste, lab adaptation) than between them.

### *Spiroplasma* and *Mesoplasma* are *T. septentrionalis* symbionts.

**FIG 4:**
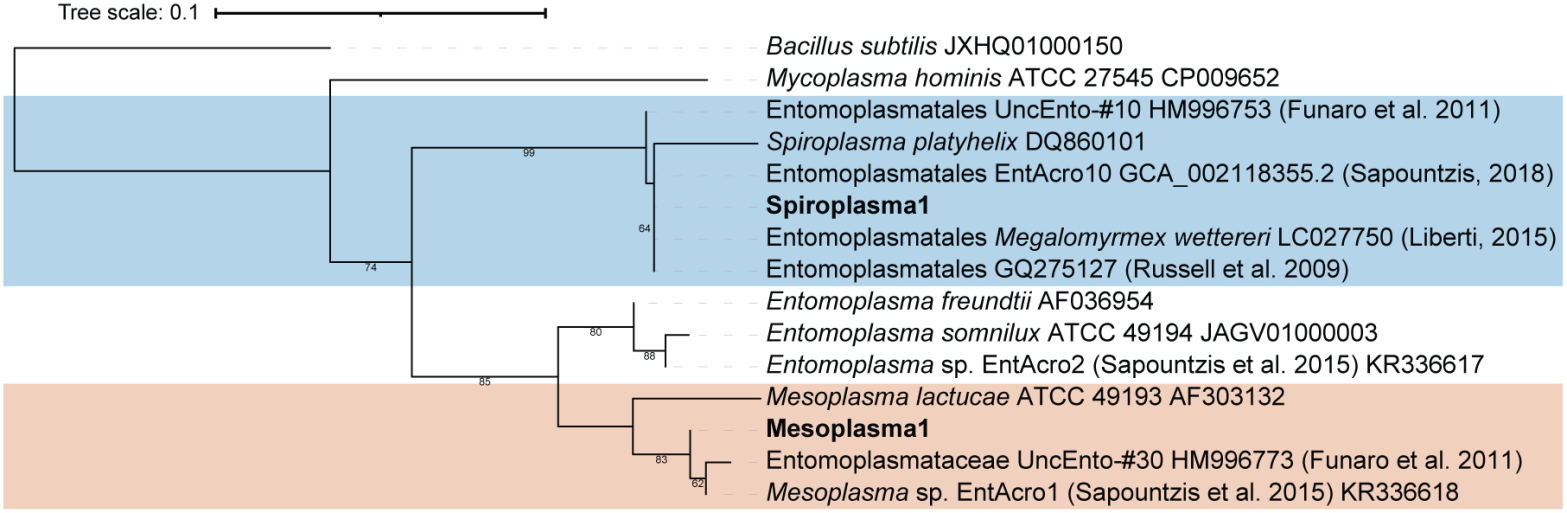
Maximum-likelihood phylogenetic tree of the dominant *Tenericutes* 16S rRNA gene ASVs from the combined multistate and two-colony data sets (in bold text) and reference 16S rRNA gene sequences sequenced from other fungus-growing ants and related *Tenericutes* strains. The tree was rooted using Bacillus subtilis and Mycoplasma hominis, and bootstrap percentages of >60 are shown.

*Mesoplasma* and *Spiroplasma* were the two dominant genera in our microbiome samples, accounting for 12% and 14% of all the reads in our data sets, respectively. These bacteria, classified within the phylum *Tenericutes*, are well-known symbionts of insects and plants (35–40). Although their function remains to be experimentally tested, *Spiroplasma* and *Mesoplasma* have been found in other fungus-growing ants (26–29, 31, 34). The most abundant *Mesoplasma* amplicon sequence variant (ASV) in our data set, amounting to 97.7% of all *Mesoplasma* reads, was highly similar to the 16S rRNA of EntAcro1 and an uncultured *Entomoplasmataceae* 16S rRNA sequence found in army ants (Fig. 4) (41). EntAcro1 is a metagenome-assembled genome (MAG) that was assembled from *Acromyrmex* fungus-growing ants (31), and related 16S rRNA sequences have also been found in *Atta columbica, Atta sexdens*, Mycetomoellerius zeteki (previously Trachymyrmex zeteki), Paratrachymyrmex cornetzi (previously Trachymyrmex cornetzi), and Cyphomyrmex rimosus fungus-growing ants. The most abundant *Spiroplasma* ASV in our data set, making up 97.3% of all *Spiroplasma* reads, was highly similar to the EntAcro10 MAG assembled from *Acromyrmex* ants (31) and for which related 16S rRNA sequences were found in *Atta*, *Acromyrmex*, and *Mycetomoellerius* (27, 29) fungus-growing ants, along with other 16S rRNA sequences that were annotated as uncultured *Entomoplasmatales* from two other fungus-growing ants, Sericomyrmex amabilis and Mycetomoellerius jamaicensis (Fig. 4) (26, 42).

**FIG 5:**
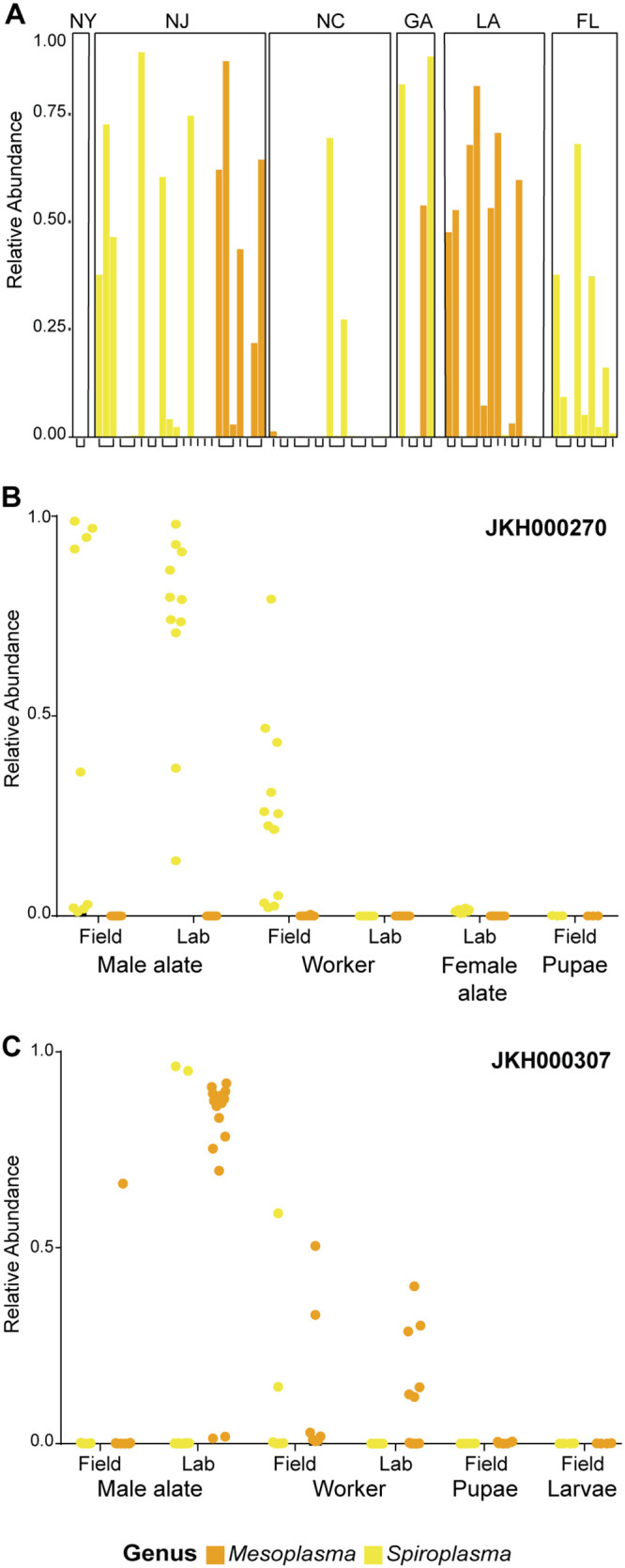
*Spiroplasma* (yellow) and *Mesoplasma* (orange) are abundant in *T. septentrionalis* ants. (A) Single bars represent the relative abundance of reads from the genus *Spiroplasma* or *Mesoplasma* in ants from the multistate data set, grouped by the state of collection. The *x* axis brackets group worker ants from the same colony. *n* = 71. (B and C) The relative abundance of the genus *Spiroplasma* or *Mesoplasma* in the two-colony data set, with each dot representing relative abundance in a single ant. All ants in this data set were colonized exclusively by either *Spiroplasma* or *Mesoplasma*.

The presence of *Mesoplasma* and *Spiroplasma* varied between different ant colonies, between ants from the same colony, and between ants from different castes, both before and after lab adaptation. In our multistate data set, colonies from New York and North Carolina had lower *Tenericutes* abundances than the other states (Fig. 5A). *Spiroplasma* was abundant in field-collected worker ants and both field-collected and lab-maintained male alates from colony JKH000270. *Mesoplasma* was abundant in field-collected and lab-maintained workers and lab-maintained male alates from colony JKH000307. In both the multistate and two-colony data sets, only one of *Spiroplasma* or *Mesoplasma* was present per colony, although not all ants from that colony were colonized by a *Tenericutes* (Fig. 5B and C). An exception to this trend was in colony JKH000307, in which *Mesoplasma* was abundant but *Spiroplasma* colonized two lab-maintained male alates and two field-collected workers. Ants colonized by *Spiroplasma* were rarely found within a colony mainly colonized by *Mesoplasma*, and vice versa (Fig. 5B and C), and no ants in our multistate or two-colony data sets were colonized by both *Spiroplasma* and *Mesoplasma.* However, in a confirmatory data set that sampled pupae from two additional ant colonies, JKH000125 and JKH000136, we found that pupae from colony JKH000136 were colonized exclusively by large amounts of *Mesoplasma* and that pupae from colony JKH000125 pupae were colonized primarily by *Spiroplasma* but also by small amounts of *Mesoplasma* (Fig. S8C). This is the only time we have observed ants colonized by both *Mesoplasma* and *Spiroplasma*.

## DISCUSSION

Our microbiome sequencing of individual *T*. *septentrionalis* ants shows that caste, lab adaptation (field-collected or lab-maintained), and the colony that an ant belongs to all influence the structure of the ant microbiome. Our multistate data set includes field-collected worker ants from 6 states representing 36 colonies, allowing us to describe a conserved microbiome of wide-ranging worker ants that contains *Thermomonas*, Escherichia, *Lautropia*, *Solirubrobacter*, *Pseudonocardia*, *Aeromicrobium*, and *Nocardioides* (Fig. 1A), all of which were prevalent in >75% of the ants in this data set. This conserved microbiome was largely confirmed in the field-collected worker ant microbiome in our two-colony data set. *Wolbachia* was also found in several samples from our multistate data set, similar to what has been documented in *Acromyrmex*, where it is thought to be transmitted maternally (28). *Mesoplasma* and *Spiroplasma* were both found throughout our data set (Fig. 5) and are also abundant in other attine ant genera (27, 28). Our results also agree with those of Ishak et al. (34), who identified 19 bacterial genera that were common in several *T. septentrionalis* colonies sampled from a single location in Texas. Of these 19 genera, 9 are among the 18 most abundant genera in our multistate data set: *Aeromicrobium*, *Nocardioides*, *Phycicoccus*, *Pseudonocardia*, *Solirubrobacter*, *Bacillus*, Pseudomonas, *Mesoplasma*, and *Spiroplasma*. The larger number of taxa found by Ishak et al. (34) is consistent with our finding that the *T. septentrionalis* ant microbiome has some biogeographic conservation, albeit limited. Colonies that are located closer together had the most similar microbiome compositions, meaning that a list of common taxa sampled from a single collection location would be larger than a conserved microbiome defined using samples collected from many different locations.

In our multistate data set, in which 1 to 3 ants were sampled per colony, the microbiomes from ants sampled from the same colony were more similar than ants sampled from different colonies. To further study the variation between the microbiomes of individual ants from a single colony, our two-colony data set included microbiomes from ants more deeply sampled from two colonies, including ants from different castes and from both before and after lab adaptation. Again, we found that ant microbiomes within colonies were more similar than those between colonies. Despite differences in their modes of microbiome transmission and selection, this bolsters a common natural pattern, e.g., where nestmates of birds (43) and mammals (44) and colonies of insects such as honey bees (9, 11) and ants (45) all have similar microbiomes. These differences that we observed between colonies could be due to local ecological factors such as regional differences in the plants, insect frass, and microbes associated with these substrates that will cause the ant microbiome to vary. The soil microbiome that surrounds the fungus garden chambers is highly conserved and overlaps minimally with the fungus garden microbiome (Lee KM and Klassen JL, unpublished data), suggesting that input from soil microbes is unlikely to drive significant differences in ant microbiomes. This is supported by our samples collected following adaptation of colonies to our lab, where environmental conditions are constant and colonies are fed only sterile cornmeal. Lab-maintained ant microbiomes are much less variable than those sampled in the field (Fig. S10B), highlighting the importance of natural foraging and environmental variation for maintaining microbiome diversity. Another possible reason why microbiomes might differ between colonies is the strong founder effects that likely occur during colony foundation. Before leaving to mate, a female alate will place a piece of the fungus garden in her infrabuccal pocket (13). After mating, this new queen will found her own colony using that piece of fungus garden, tending it and producing all offspring in that colony. The microbiome possessed by the queen will be passed to these workers, who will thereafter pass it on to subsequent generations of workers, thus creating a bottleneck in microbiome composition caused by the limited microbial diversity possessed by the queen during colony foundation.

Within a colony, microbiome composition depended on ant castes, which determined the greatest amount of variation between microbiome composition in the two-colony data set (Fig. 3B). Gut microbiomes often differ between social insect castes, likely due to the specialized functional role played by each caste (2). Each insect in a colony will shed their gut lining during pupation, thereby also shedding their gut microbiome and requiring the gut microbiome to then reassemble. The microbiomes of reproductive castes often differ from those of the worker castes in termites (8), honey bees (9), and other species of ants (45). In *T. septentrionalis* colonies, workers forage for plant material and caterpillar frass that have their own microbiomes, and these bacteria might be transferred to the workers and to the fungus garden that they tend. Such microbes brought into the colony via foraging could then be transferred to all ant microbiomes in that colony. However, such transfer may be rare due to the ants grooming themselves, other ants, and the fungus garden to remove pathogens (46). In contrast, the role of male and female alates is to mate and, for females, to start a new colony. The different microbiomes possessed by these castes could therefore reflect their need to reproduce, which the workers do not experience. Female alates possess large amounts of lipids, proteins, and carbohydrates to provide energy when they start their colonies (47), and male alates must travel further than female alates to mate (48). The different microbiome composition possessed by male and female alates (Fig. 3C) could therefore reflect the differing energy and nutrient requirements of their different reproductive behaviors (47, 48). Although we show that *T. septentrionalis* microbiomes differ between castes, how vertical versus environmental transmission contributes to these differences remains unknown.

The presence and abundance of *Mesoplasma* and *Spiroplasma* differ between each colony and individual ant, explaining some of the differences in ant microbiome composition between ant colonies and castes. The most abundant *Mesoplasma* and *Spiroplasma* 16S rRNA genes detected in our data set are very similar to those of other *Tenericutes* found in other fungus-growing ant genera, which suggests that these bacteria are common members of the fungus-growing ant symbiosis. The functional role played by these *Tenericutes* is unknown. They are unlikely to be reproductive pathogens, based on their high abundances in worker ants, which are reproductive dead ends. *Acromyrmex* and *Atta* callows (i.e., newly emerged worker ants) were readily colonized by *Tenericutes* when cared for by other *Tenericutes*-containing worker ants, but not after 21 days in the absence of such ants (28). Thus, *Tenericutes* symbionts are likely transferred between ants via care from other workers. Fluorescent *in situ* hybridization (FISH) imaging has also been used to localize *Tenericutes* to ant guts but not in reproductive organs. Because we only have characterized female alate microbiomes from a single colony, future studies should include a wider sampling of this caste. *Tenericutes* might also be acquired from an environmental source (e.g., foraged plants or caterpillar frass), or during interactions with other ant colonies (49).

*Tenericutes* symbionts of fungus-growing ants may be nutritional symbionts. Metagenomic reconstructions of *Spiroplasma* and *Mesoplasma* symbionts from *Acromyrmex* ants suggested that these bacteria may decompose excess arginine, and, for *Mesoplasma*, citrate (31). Arginine recycling would produce ammonium that could be provided to the fungus garden via ant fecal droplets, and the degradation of citrate, acquired from foraged fruit, leaves, and other plant material, could produce acetate for use by the ants. If these hypotheses were supported, it would suggest that *Tenericutes* provide nutrients for both the ants and the cultivar fungus. In the phylogeny of fungus-growing ants, *Trachymyrmex* is the closest phylogenetic neighbor to the leaf-cutting ants (14, 50, 51). The fact that both the *Mesoplasma* and *Spiroplasma* found in this study are closely related to those found in *Atta* and *Acromyrmex*, unlike in more basal fungus-growing ant taxa, reflects this phylogenetic relationship. However, in our data, the presence of *Tenericutes* varied extensively between ant colonies and castes (Fig. 5), meaning that these symbionts must not be absolutely required for ant survival, especially given their complete absence in some ants, a hypothesis to be tested in future studies. Instead, such extreme variation in abundance between colonies and individual ants could imply that *Mesoplasma* and *Spiroplasma* are ant pathogens or commensals, at least in some ecological contexts. Interestingly, we did not find any coinfections of both *Mesoplasma* and *Spiroplasma* together in the same ant, except for in pupae from a single ant colony (Fig. S8C). This might suggest a transmission bottleneck during microbiome assembly that allows for colonization of only one of these symbionts or competition between *Spiroplasma* and *Mesoplasma* in these ants.

Our study shows how microbiome assembly differs between ant colonies and castes and as a result of lab adaptation. *T. septentrionalis* ant microbiomes did not vary extensively between sampling locations but did vary substantially between ant colonies and castes. Lab adaptation showed the importance of ant foraging or environmental variation for maintaining microbiome diversity, with lab-maintained microbiomes being less variable than field-collected ones as a reflection of their more stable environment. The presence of *Mesoplasma* and *Spiroplasma* causes some of these differences between colonies and castes and as the result of lab-adaptation. Together, these data show how the composition of a social insect microbiome is the result of colony- and caste-specific factors.

## MATERIALS AND METHODS

### Sample collection.

*Trachymyrmex septentrionalis* colonies were collected in New Jersey, New York, North Carolina, Florida, Georgia, and Louisiana during 2014 to 2018 (supplemental File 1, available at https://github.com/klassen-lab/Green_2022; Fig. S1). Permits for *T. septentrionalis* collection include the New Jersey Department of Environmental Protection Division of Parks and Forestry State Park Service unnumbered letters of authorization, New York State Department of Environmental Conservation license to collect or possess: scientific no. 915, permit for research in Suffolk County, North Carolina Division of Parks and Recreation scientific collection and research permit 2015_0030, Florida Department of Agriculture and Consumer Services unnumbered letters of authorization, Georgia Department of Natural Resources State Parks and Historic Sites scientific research and collection permit 032015, and Louisiana Department of Wildlife and Fisheries permit WL-Research2016-10, in addition to permits from the U.S. Department of Agriculture for the transportation of ants to the University of Connecticut (USDA; P526P-14-00684). Freshly collected ants (here, “field-collected ants”) were stored in DESS (20% dimethyl sulfoxide, 250 mM disodium EDTA, and saturated sodium chloride) (52) on dry ice immediately after colony collection in the field and then stored at −80°C in the lab until sampling for DNA extraction. The remainder of each colony (including both fungus gardens and the remaining ants) was brought back to the University of Connecticut, kept in a USDA-approved and temperature-controlled room, and maintained following Sosa-Calvo et al. (53). Colonies were placed in a 6 and 3/4-in. long by 4 and 13/16-in. wide by 2 and 3/8-in. high box that was lined with plaster of paris, watered biweekly to maintain humidity, and provided with sterile cornmeal *ad libitum* as food for the fungus garden. Ants sampled from these colonies are referred to as “lab-maintained.”

We generated two major data sets for this study. The first, which we refer to as the “multistate” data set, included field-collected workers from all of the states mentioned above. This data set was designed to test if ant microbiomes varied throughout the ants’ geographic range. The second, which we refer to as the “two-colony” data set, was made up of multiple ants sampled from colony JKH000270, collected from Friendship, NJ, in July 2017, and colony JKH000307, collected from Quaker Bridge, NJ, in July 2018. We sampled all available castes and life stages, including workers, pupae, larvae, male alates, and female alates, to test if microbiomes differed between castes and following lab adaptation. The colonies in the two-colony data set were selected based on the availability of matching field and lab samples. Colony JKH000270 lab-maintained ants were sampled after 1 year and 4 months (some male alates were sampled earlier), and colony JKH000307 lab-maintained ants were sampled after 4 months. Worker ants collected from the lab-maintained colonies were sampled immediately before DNA extraction without storage in DESS. Although lab-maintained samples were not stored in DESS, our previous research demonstrating that DESS effectively preserves the fungus garden microbiome suggests that this is unlikely to be a significant source of bias (52). Two confirmatory data sets were also used, with one including pupae and another including dissected ant guts and whole ants sampled from the same colony (Fig. S2 and S8).

10.1128/msphere.00989-21.2 FIG S2 Comparison of whole-ant and gut microbiomes. (A) The phyla present in whole-ant and ant gut microbiomes at >15% abundance. Single bars represent individual ants. The phyla are differentiated by color, with “other” representing phyla present at <15% relative abundance. (B and C) PCoA ordinations of weighted (B) and unweighted (C) Unifrac distances between whole-ant and ant gut microbiomes. Sample types are indicated using color. *n* = 10. WUF PERMANOVA: R^2^ = 0.342, *P* = 0.01; UUF PERMANOVA: R^2^ = 0.110, *P* = 0.451; Bray-Curtis PERMANOVA: R^2^ = 0.352, *P* = 0.053. Download FIG S2, TIF file, 1.4 MB. Copyright © 2022 Green and Klassen. 2022 Green and Klassen. https://creativecommons.org/licenses/by/4.0/ This content is distributed under the terms of the Creative Commons Attribution 4.0 International license.

### Sample preparation and DNA extraction.

All ants were surface-cleaned using a 10-s submersion in 70% ethanol followed by a 10-s submersion in phosphate-buffered saline (PBS), repeated three times (54). Some pupae were ethanol-washed and others were not to determine if the *Tenericutes* symbionts were located on their exoskeleton or internally. DNA was extracted from these surface-cleaned ants using the bead-beating and chloroform-isopropanol protocol described previously (52, 55). Our preliminary data showed minimal differences between microbiomes generated using whole, surface-cleaned ants and ant guts, so we used whole, surface-cleaned ants throughout this study for simplicity (Fig. S2) while recognizing that actinobacteria in our data set (predominantly *Phycicoccus* and *Pseudonocardia*) may be present due to incomplete surface cleaning. Negative controls containing only the DNA extraction reagents were processed alongside each batch of ant samples. The DNA concentration of each extract and negative control was determined using the Qubit double-stranded DNA (dsDNA) high-sensitivity assay protocol and a Qubit 3.0 fluorimeter (Invitrogen, Carlsbad, CA).

### PCR screening and community amplicon sequencing.

DNA samples were PCR amplified using primers 515F and 806R to amplify the 16S rRNA gene V4 region (56). A total of 10 ng of template DNA was added to 5 μL green GoTaq reaction mix buffer (Promega, Madison, WI), 1.25 units GoTaq DNA polymerase (Promega), 10 μmol of each primer, and 300 ng bovine serum albumin (BSA; New England BioLabs, Inc., Ipswitch, MA), to which nuclease-free H_2_O was added to reach a volume of 25 μL. Thermocycler conditions (Bio-Rad, Hercules, CA) were 3 min at 95°C, 30 cycles of 30 sec at 95°C, 30 sec at 50°C, and 60 sec at 72°C, followed by a 5-min cycle at 72°C and then an indefinite hold at 4°C. Gel electrophoresis confirmed the expected band size of 300 to 350 bp.

Samples that produced PCR products of the expected size and all negative controls were prepared for community amplicon sequencing of the 16S rRNA gene V4 region using an Illumina MiSeq instrument at the University of Connecticut Microbial Analysis, Resources, and Services facility. Approximately 30 ng of DNA from each sample was added to a 96-well plate containing 10 μmol each of Illumina-barcoded versions of primers 515F and 806R, 5 μL AccuPrime buffer (Invitrogen, Carlsbad, CA), 50 mM MgSO_4_ (Invitrogen), 300 ng BSA (New England BioLabs, Inc., Ipswitch, MA), a 1-μmol spike-in of both nonbarcoded primers 515F and 806R, and 1 unit AccuPrime polymerase (Invitrogen), to which nuclease-free H_2_O was added to a volume of 50 μL. Reaction mixes were separated in a 384-well plate using an epMotion 5075 liquid-handling robot (Eppendorf, Hamburg, Germany), forming three replicates (each with a volume of 16.7 μL). This plate was then transferred to a thermocycler (Eppendorf), which used the following conditions: 2 min at 95°C, 30 cycles of 15 sec at 95°C, 60 sec at 55°C, and 60 sec at 68°C, a final extension for 5 min at 68°C, and an indefinite hold at 4°C. Post-PCR, triplicate reactions were repooled using the epMotion robot, and DNA concentrations were quantified using a QIAxcel Advanced capillary electrophoresis system (Qiagen, Hilden, Germany). Samples with concentrations of >0.5 ng/μL were pooled using equal DNA masses to create the final sequencing libraries. Libraries were then bead-cleaned using Mag-Bind RXNPure plus beads (Omega, Norcross, GA) in a 1:0.8 ratio of sequencing library to bead volume. Cleaned library pools were adjusted to a concentration of 1.1 ng/μL ± 0.1 ng/μL, and their concentrations were confirmed using the Qubit dsDNA high-sensitivity assay on a Qubit 3.0 fluorimeter (Invitrogen). Microbial community sequencing on an Illumina MiSeq instrument using 2 × 250-bp libraries (Illumina, San Diego, CA) was completed in 4 batches, each containing either the multistate data set (104 samples plus 5 negative controls), the two-colony data set (147 samples plus 9 negative controls), the pupae data set (20 samples plus 1 negative control), or the whole ant/gut dissection data set (10 samples plus 1 negative control).

### Bioinformatic analyses.

We analyzed all data sets individually. Reads were analyzed using R v3.5.3 (57) and the DADA2 v1.11.1 (58) pipeline for amplicon sequence variants (ASVs) (https://benjjneb.github.io/dada2/index.html, accessed 11 November 2017). Read counts per sample were 5 to 1,952,121 (multistate data set), 34 to 212,473 (two-colony data set), 15,495 to 92,408 (pupae data set), and 17,565 to 132,790 (whole-ant/gut dissection data set; supplemental File 1, https://github.com/klassen-lab/Green_2022). Metadata files were imported into phyloseq v1.26.1 (59), creating a phyloseq R object that was used for subsequent analyses. Reads that were not classified as belonging to the kingdom *Bacteria* (i.e., those identified as *Archaea* or *Eukaryote*) using the SILVA database v128 (60, 61) were removed. The ASVs that matched mitochondria were then removed separately because SILVA included them in the kingdom *Bacteria*. Samples were screened for contamination using the decontam v1.2.1 (62) prevalence protocol with a default threshold value of 0.1. No reads were flagged as contaminants for any of the data sets, resulting in 1,798, 1,353, 470, and 161 unique ASVs for the multistate, two-colony, pupae, and whole-ant/gut dissection data sets, respectively (supplemental File 1, https://github.com/klassen-lab/Green_2022). Negative-control samples were not considered further. All samples were rarefied to 20,000 reads for the multistate data set, 5,000 reads for the two-colony data set, 10,000 reads for the pupae data set, and 14,500 reads for the whole-ant/gut dissection data set, and read counts were converted to relative abundances. The final phyloseq objects contained 71, 110, 19, and 10 samples for the multistate, two-colony, pupae, and whole-ant/gut dissection data sets, respectively.

Alpha diversity (Shannon and Simpson metrics) was measured using the phyloseq plot_richness command and compared using the R stats aov and TukeyHSD commands. Beta diversity was measured using weighted Unifrac (WUF) and unweighted Unifrac (UUF) distance metrics. Weighted and unweighted Unifrac distances were calculated, ordinated, and viewed using the distance, ordinate, and plot_ordinate phyloseq commands, respectively. Ellipses were added to the PCoA plots of each Unifrac distance using the ggplot2 (63) command stat_ellipse with a default multivariate *t*-distribution. PERMANOVA tests were calculated using vegan v2.5-4 (64). Although weighted Unifrac and unweighted Unifrac distances were used for each test, to keep the text concise, only one test is listed in the text, and the complementary values are presented in the Supplementary Figures. After exporting the multistate phyloseq object into Microsoft Excel, we chose the first sample from each colony to be used in the Mantel tests, creating a new data set containing 36 samples. Each sample and its corresponding GPS locations were uploaded to GeoMatrix (65) (Geographic Distance Matrix Generator v1.2.3) following the specified format. The GeoMatrix output was downloaded and imported into R. Full Mantel tests were completed using the GeoMatrix file and the WUF and UUF distance matrices for the 36 samples that included only one ant from each colony using the vegan command mantel. Partial Mantel tests were completed, and Mantel correlograms were created using the vegan mantel.correlog and plot commands, respectively. Kolmogorov-Smirnov and Wilcoxon rank sum tests were completed using the R commands ks.test and wilcox.test, respectively. If the *P* value for the Kolmogorov-Smirnov test was >0.05, then the Wilcoxon rank sum tests were used to compare the distributions of Unifrac distances; otherwise, a *t* test was used (Fig. S10B). A heatmap of the bacterial genera in the two-colony data set whose read abundances were >5% was created using the heatmap.2 command in gplots v3.0.3 (66). Heatmaps describing the median relative abundances of genera with a read abundance of >5% within workers and male alates from colonies JKH000270 and JKH00307 were created using Charticulator v2.0.4 (https://charticulator.com/app/index.html).

A phylogenetic tree was constructed to show relationships between the 16S rRNA sequences of the *Tenericutes* generated during this study and those that had been previously isolated from the leaf-cutting ants *Atta* and *Acromyrmex*. The most abundant *Spiroplasma* ASV and *Mesoplasma* ASV from the combined multistate and two-colony data sets were aligned with reference *Tenericutes* sequences generated from other ants and related *Mesoplasma* and *Spiroplasma* sequences, all downloaded from NCBI, using MUSCLE v3.8.31 (67), and then trimmed to the same alignment length. A phylogenetic tree, rooted using the Bacillus subtilis 16S rRNA gene sequence, was calculated using a GTRGAMMAI substitution model and 500 bootstrap replicates in RAxML v8.2.11 (68).

### Data availability.

The commands used for all analyses are attached as supplemental File 2, available at https://github.com/klassen-lab/Green_2022. All raw sequencing reads are deposited in the NCBI database under BioProject PRJNA687229.

## References

[1] The Editors of Encyclopedia Britannica. 2018. Social insect. Encyclopædia Britannica, Inc., Chicago, IL.

[2] Sinotte VM, Renelies-Hamilton J, Taylor BA, Ellegaard KM, Sapountzis P, Vasseur-Cognet M, Poulsen M. 2020. Synergies between division of labor and gut microbiomes of social insects. Front Ecol Evol 7:e00503. doi:10.3389/fevo.2019.00503.

[3] Hongoh Y, Ekpornprasit L, Inoue T, Moriya S, Trakulnaleamsai S, Ohkuma M, Noparatnaraporn N, Kudo T. 2006. Intracolony variation of bacterial gut microbiota among castes and ages in the fungus-growing termite *Macrotermes gilvus*. Mol Ecol 15:505–516. doi:10.1111/j.1365-294X.2005.02795.x. 16448416

[4] Schnorr SL, Hofman CA, Netshifhefhe SR, Duncan FD, Honap TP, Lesnik J, Lewis CM. 2019. Taxonomic features and comparisons of the gut microbiome from two edible fungus-farming termites (*Macrotermes falciger*; *M. natalensis*) harvested in the Vhembe district of Limpopo, South Africa. BMC Microbiol 19:e164. doi:10.1186/s12866-019-1540-5. PMC6637627 31315576

[5] Dietrich C, Kohler T, Brune A. 2014. The cockroach origin of the termite gut microbiota: patterns in bacterial community structure reflect major evolutionary events. Appl Environ Microbiol 80:2261–2269. doi:10.1128/AEM.04206-13. 24487532 PMC3993134

[6] Otani S, Mikaelyan A, Nobre T, Hansen LH, Koné NA, Sørensen SJ, Aanen DK, Boomsma JJ, Brune A, Poulsen M. 2014. Identifying the core microbial community in the gut of fungus-growing termites. Mol Ecol 23:4631–4644. doi:10.1111/mec.12874. 25066007

[7] Otani S, Hansen LH, Sorensen SJ, Poulsen M. 2016. Bacterial communities in termite fungus combs are comprised of consistent gut deposits and contributions from the environment. Microb Ecol 71:207–220. doi:10.1007/s00248-015-0692-6. 26518432 PMC4686563

[8] Otani S, Zhukova M, Koné NA, da Costa RR, Mikaelyan A, Sapountzis P, Poulsen M. 2019. Gut microbial compositions mirror caste-specific diets in a major lineage of social insects. Environ Microbiol Rep 11:196–205. doi:10.1111/1758-2229.12728. 30556304 PMC6850719

[9] Kapheim KM, Rao VD, Yeoman CJ, Wilson BA, White BA, Goldenfeld N, Robinson GE. 2015. Caste-specific differences in hindgut microbial communities of honey bees (*Apis mellifera*). PLoS One 10:e0123911. doi:10.1371/journal.pone.0123911. 25874551 PMC4398325

[10] Kwong WK, Moran NA. 2016. Gut microbial communities of social bees. Nat Rev Microbiol 14:374–384. doi:10.1038/nrmicro.2016.43. 27140688 PMC5648345

[11] Moran NA, Hansen AK, Powell JE, Sabree ZL. 2012. Distinctive gut microbiota of honey bees assessed using deep sampling from individual worker bees. PLoS One 7:e36393. doi:10.1371/journal.pone.0036393. 22558460 PMC3338667

[12] Wilson EO. 1971. The insect societies. Harvard University Press, Cambridge, MA.

[13] Mueller UG, Schultz TR, Currie CR, Adams RMM, Malloch D. 2001. The origin of the attine ant-fungus mutualism. Q Rev Biol 76:169–197. doi:10.1086/393867. 11409051

[14] Schultz TR, Brady SG. 2008. Major evolutionary transitions in ant agriculture. Proc Natl Acad Sci USA 105:5435–5440. doi:10.1073/pnas.0711024105. 18362345 PMC2291119

[15] Weber NA. 1972. The fungus-culturing behavior of ants. Am Zool 12:577–587. doi:10.1093/icb/12.3.577.

[16] Hölldobler B, Wilson EO. 1990. The ants. Harvard University Press, Cambridge, MA.

[17] Solomon SE, Rabeling C, Sosa-Calvo J, Lopes CT, Rodrigues A, Vasconcelos HL, Bacci M, Mueller UG, Schultz TR. 2019. The molecular phylogenetics of *Trachymyrmex* Forel ants and their fungal cultivars provide insights into the origin and coevolutionary history of ‘higher-attine’ ant agriculture. Syst Entomol 44:939–956. doi:10.1111/syen.12370.

[18] Wheeler WM. 1907. The fungus-growing ants of North America. Bull Am Museum Nat Hist 23:669–807.

[19] Munkacsi AB, Pan JJ, Villesen P, Mueller UG, Blackwell M, McLaughlin DJ. 2004. Convergent coevolution in the domestication of coral mushrooms by fungus-growing ants. Proc Biol Sci 271:1777–1782. doi:10.1098/rspb.2004.2759. 15315892 PMC1691797

[20] Weber NA. 1966. Fungus-growing ants. Science 153:587–604. doi:10.1126/science.153.3736.587. 17757227

[21] De Fine Licht HH, Boomsma JJ. 2010. Forage collection, substrate preparation, and diet composition in fungus-growing ants. Ecol Entomol 35:259–269. doi:10.1111/j.1365-2311.2010.01193.x.

[22] Currie CR, Scott JA, Summerbell RC, Malloch D. 1999. Fungus-growing ants use antibiotic-producing bacteria to control garden parasites. Nature 398:701–705. doi:10.1038/19519.

[23] Goldstein SL, Klassen JL. 2020. *Pseudonocardia* symbionts of fungus-growing ants and the evolution of defensive secondary metabolism. Front Microbiol 11:e621041. 10.3389/fmicb.2020.621041 PMC7793712 33424822

[24] Klassen JL. 2014. Microbial secondary metabolites and their impacts on insect symbioses. Curr Opin Insect Sci 4:15–22. doi:10.1016/j.cois.2014.08.004. 28043403

[25] Van Borm S, Billen J, Boomsma JJ. 2002. The diversity of microorganisms associated with *Acromyrmex* leafcutter ants. BMC Evol Biol 2:9. doi:10.1186/1471-2148-2-9. 12019020 PMC113273

[26] Liberti J, Sapountzis P, Hansen LH, Sørensen SJ, Adams RMM, Boomsma JJ. 2015. Bacterial symbiont sharing in *Megalomyrmex* social parasites and their fungus-growing ant hosts. Mol Ecol 24:3151–3169. doi:10.1111/mec.13216. 25907143 PMC5008137

[27] Sapountzis P, Zhukova M, Hansen LH, Sørensen SJ, Schiøtt M, Boomsma JJ. 2015. *Acromyrmex* leaf-cutting ants have simple gut microbiota with nitrogen-fixing potential. Appl Environ Microbiol 81:5527–5537. doi:10.1128/AEM.00961-15. 26048932 PMC4510174

[28] Zhukova M, Sapountzis P, Schiøtt M, Boomsma JJ. 2017. Diversity and transmission of gut bacteria in *Atta* and *Acromyrmex* leaf-cutting ants during development. Front Microbiol 8:e01942. 10.3389/fmicb.2017.01942 PMC5641371 29067008

[29] Sapountzis P, Nash DR, Schiøtt M, Boomsma JJ. 2019. The evolution of abdominal microbiomes in fungus-growing ants. Mol Ecol 28:879–899. doi:10.1111/mec.14931. 30411820 PMC6446810

[30] Zani RDOA, Ferro M, Bacci M. 2021. Three phylogenetically distinct and culturable diazotrophs are perennial symbionts of leaf-cutting ants. Ecol Evol 11:17686–17699. doi:10.1002/ece3.8213. 35003632 PMC8717316

[31] Sapountzis P, Zhukova M, Shik JZ, Schiott M, Boomsma JJ. 2018. Reconstructing the functions of endosymbiotic mollicutes in fungus-growing ants. Elife 7:e39209. doi:10.7554/eLife.39209. 30454555 PMC6245734

[32] Rabeling C, Cover SP, Johnson RA, Mueller UG. 2007. A review of the North American species of the fungus-gardening ant genus *Trachymyrmex* (Hymenoptera: Formicidae). Zootaxa 1664:e180014. https://www.mapress.com/zt/article/view/zootaxa.1664.1.1.

[33] Seal JN, Brown L, Ontiveros C, Thiebaud J, Mueller UG. 2015. Gone to Texas: phylogeography of two *Trachymyrmex* (Hymenoptera: Formicidae) species along the southeastern coastal plain of North America. Biol J Linn Soc Lond 114:689–698. doi:10.1111/bij.12426.

[34] Ishak HD, Miller JL, Sen R, Dowd SE, Meyer E, Mueller UG. 2011. Microbiomes of ant castes implicate new microbial roles in the fungus-growing ant *Trachymyrmex septentrionalis*. Sci Rep 1:e00204. doi:10.1038/srep00204. PMC3244503 22355719

[35] Hackett KJ, Whitcomb RF, Tully JG, Lloyd JE, Anderson JJ, Henegar RB, Rose DL, Clark EA, Vaughn JL. 1992. Lampyridae (Coleoptera): a plethora of mollicute associations. Microb Ecol 23:181–193. doi:10.1007/BF00172639. 24192863

[36] Ammar E-D, Hogenhout SA. 2006. Mollicutes associated with arthropods and plants, p 97–118. *In* Bourtzis K, Miller TA (ed), Insect symbiosis. CRC Press, Boca Raton, FL.

[37] 2015. Bergey’s manual of systematics of archaea and bacteria. Bergey’s Manual Trust, Hoboken, NJ.

[38] Williamson DL, Adams JR, Whitcomb RF, Tully JG, Carle P, Konai M, Bove JM, Henegar RB. 1997. *Spiroplasma platyhelix* sp. nov., a new mollicute with unusual morphology and genome size from the dragonfly *Pachydiplax longipennis*. Int J Syst Bacteriol 47:763–766. doi:10.1099/00207713-47-3-763. 9226909

[39] Rose DL, Kocka JP, Somerson NL, Tully JG, Whitcomb RF, Carle P, Bove JM, Colflesh DE, Williamson DL. 1990. *Mycoplasma lactucae* sp. nov., a sterol-requiring Mollicute from a plant surface. Int J Syst Bacteriol 40:138–142. doi:10.1099/00207713-40-2-138. 2223606

[40] Tully JG, Whitcomb RF, Hackett KJ, Rose DL, Henegar RB, Bove JM, Carle P, Williamson DL, Clark TB. 1994. Taxonomic descriptions of eight new non-sterol-requiring mollicutes assigned to the genus *Mesoplasma*. Int J Syst Bacteriol 44:685–693. doi:10.1099/00207713-44-4-685. 7726910

[41] Funaro CF, Kronauer DJC, Moreau CS, Goldman-Huertas B, Pierce NE, Russell JA. 2011. Army ants harbor a host-specific clade of *Entomoplasmatales* bacteria. Appl Environ Microbiol 77:346–350. doi:10.1128/AEM.01896-10. 21075876 PMC3019723

[42] Russell JA, Moreau CS, Goldman-Huertas B, Fujiwara M, Lohman DJ, Pierce NE. 2009. Bacterial gut symbionts are tightly linked with the evolution of herbivory in ants. Proc Natl Acad Sci USA 106:21236–21241. doi:10.1073/pnas.0907926106. 19948964 PMC2785723

[43] White J, Mirleau P, Danchin E, Mulard H, Hatch SA, Heeb P, Wagner RH. 2010. Sexually transmitted bacteria affect female cloacal assemblages in a wild bird. Ecol Lett 13:1515–1524. doi:10.1111/j.1461-0248.2010.01542.x. 20961376 PMC3772342

[44] Leclaire S, Nielsen JF, Drea CM. 2014. Bacterial communities in meerkat anal scent secretions vary with host sex, age, and group membership. Behav Ecol 25:996–1004. doi:10.1093/beheco/aru074.

[45] Segers FHID, Kaltenpoth M, Foitzik S. 2019. Abdominal microbial communities in ants depend on colony membership rather than caste and are linked to colony productivity. Ecol Evol 9:13450–13467. doi:10.1002/ece3.5801. 31871657 PMC6912891

[46] Currie C, Stuart A. 2001. Weeding and grooming of pathogens in agriculture by ants. Proc R Soc Lond B 268:1033–1039. doi:10.1098/rspb.2001.1605. PMC1088705 11375087

[47] Seal JN, Tschinkel WR. 2007. Energetics of newly-mated queens and colony founding in the fungus-gardening ants *Cyphomyrmex rimosus* and *Trachymyrmex septentrionalis* (Hymenoptera: Formicidae). Physiol Entomol 32:8–15. doi:10.1111/j.1365-3032.2006.00534.x.

[48] Matthews AE, Kellner K, Seal JN. 2021. Male-biased dispersal in a fungus-gardening ant symbiosis. Ecol Evol 11:2307–2320. doi:10.1002/ece3.7198. 33717457 PMC7920773

[49] Howe J, Schiøtt M, Boomsma JJ. 2019. Horizontal partner exchange does not preclude stable mutualism in fungus-growing ants. Behav Ecol 30:372–382. doi:10.1093/beheco/ary176.

[50] Sosa-Calvo J, Fernández F, Schultz TR. 2019. Phylogeny and evolution of the cryptic fungus-farming ant genus Myrmicocrypta F. Smith (Hymenoptera: Formicidae) inferred from multilocus data. Syst Entomol 44:139–162. doi:10.1111/syen.12313.

[51] Branstetter MG, Ješovnik A, Sosa-Calvo J, Lloyd MW, Faircloth BC, Brady SG, Schultz TR. 2017. Dry habitats were crucibles of domestication in the evolution of agriculture in ants. Proc R Soc B 284:e20170095. doi:10.1098/rspb.2017.0095. PMC5394666 28404776

[52] Lee KM, Adams M, Klassen JL. 2019. Evaluation of DESS as a storage medium for microbial community analysis. PeerJ 7:e6414. doi:10.7717/peerj.6414. 30740279 PMC6368006

[53] Sosa-Calvo J, Jesovnik A, Okonski E, Schultz TR. 2015. Locating, collecting, and maintaining colonies of fungus-farming ants (Hymenoptera: Formicidae: Myrmicinae: Attini). Sociobiology 62:300–320. doi:10.13102/sociobiology.v62i2.300-320.

[54] Sanders JG, Powell S, Kronauer DJC, Vasconcelos HL, Frederickson ME, Pierce NE. 2014. Stability and phylogenetic correlation in gut microbiota: lessons from ants and apes. Mol Ecol 23:1268–1283. doi:10.1111/mec.12611. 24304129

[55] Green EA, Smedley SR, Klassen JL. 2021. North American fireflies host low bacterial diversity. Microb Ecol 82:793–804. doi:10.1007/s00248-021-01718-7. 33609143

[56] Caporaso JG, Lauber CL, Walters WA, Berg-Lyons D, Lozupone CA, Turnbaugh PJ, Fierer N, Knight R. 2011. Global patterns of 16S rRNA diversity at a depth of millions of sequences per sample. Proc Natl Acad Sci USA 108:4516–4522. doi:10.1073/pnas.1000080107. 20534432 PMC3063599

[57] R Core Team. 2018. R: a language and environment for statistical computing. R Foundation for Statistical Computing, Vienna, Austria.

[58] Callahan BJ, McMurdie PJ, Rosen MJ, Han AW, Johnson AJA, Holmes SP. 2016. : DADA2: high-resolution sample inference from Illumina amplicon data. Nat Methods 13:581–583. doi:10.1038/nmeth.3869. 27214047 PMC4927377

[59] McMurdie PJ, Holmes S. 2013. phyloseq: an R package for reproducible interactive analysis and graphics of microbiome census data. PLoS One 8:e61217. doi:10.1371/journal.pone.0061217. 23630581 PMC3632530

[60] Quast C, Pruesse E, Yilmaz P, Gerken J, Schweer T, Yarza P, Peplies J, Glöckner FO. 2013. The SILVA ribosomal RNA gene database project: improved data processing and web-based tools. Nucleic Acids Res 41:590–596. doi:10.1093/nar/gks1219. PMC3531112 23193283

[61] Yilmaz P, Parfrey LW, Yarza P, Gerken J, Pruesse E, Quast C, Schweer T, Peplies J, Ludwig W, Glöckner FO. 2014. The SILVA and “all-species Living Tree Project (LTP)” taxonomic frameworks. Nucleic Acids Res 42:643–648. doi:10.1093/nar/gkt1209. 24293649 PMC3965112

[62] Davis NM, Proctor DM, Holmes SP, Relman DA, Callahan BJ. 2018. Simple statistical identification and removal of contaminant sequences in marker-gene and metagenomics data. Microbiome 6:226. doi:10.1186/s40168-018-0605-2. 30558668 PMC6298009

[63] Wickham H. 2016. ggplot2: elegant graphics for data analysis. Springer-Verlag, New York, NY.

[64] Oksanen J, Blanchet FG, Friendly M, Kindt R, Pierre L, McGlinn DPR, Minchin RB, O’Hara Gls Solymos P, Stevens MHH, Szoecs E, Wagner H. 2019. vegan: community ecology package. R package version 2.5-4. https://cran.r-project.org/web/packages/vegan/vegan.pdf

[65] Ersts PJ. 2021. Geographic Distance Matrix Generator (version 1.2.3). Accessed 2021-02-08. http://biodiversityinformatics.amnh.org/open_source/gdmg.

[66] Warnes GR, Bolker B, Bonebakker L, Gentleman R, Huber W, Liaw A, Lumley T, Maechler M, Magnusson A, Moeller S, Schwartz M, Venables B. 2009. gplots: various R programming tools for plotting data. https://cran.r-project.org/web/packages/gplots/index.html.

[67] Edgar RC. 2004. MUSCLE: multiple sequence alignment with high accuracy and high throughput. Nucleic Acids Res 32:1792–1797. doi:10.1093/nar/gkh340. 15034147 PMC390337

[68] Stamatakis A. 2014. RAxML version 8: a tool for phylogenetic analysis and post-analysis of large phylogenies. Bioinformatics 30:1312–1313. doi:10.1093/bioinformatics/btu033. 24451623 PMC3998144

