# Supplementary figures and images for: Trachymyrmex septentrionalis Ant Microbiome Assembly Is Unique to Individual Colonies and Castes
Source: mSphere. 2022 Jul 7;7(4):e00989-21. doi: 10.1128/msphere.00989-21 (PMC9429924; doi:10.1128/msphere.00989-21)

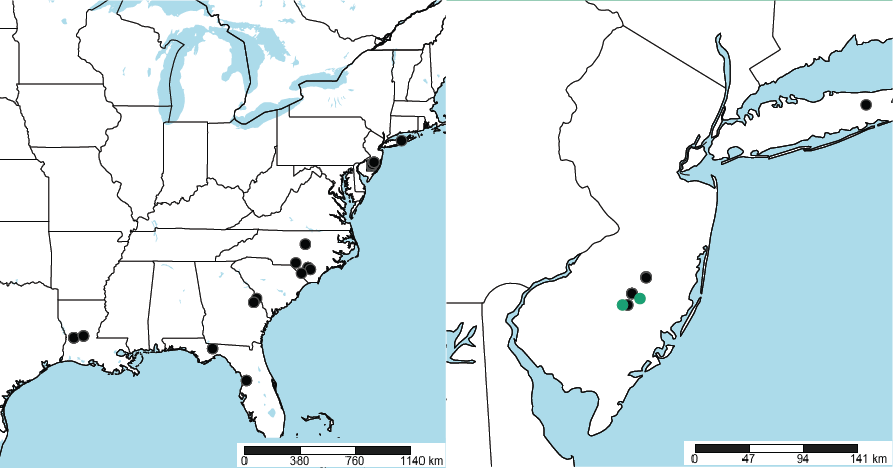

Supplement: FIG S1 [file msphere.00989-21-sf001.tif]

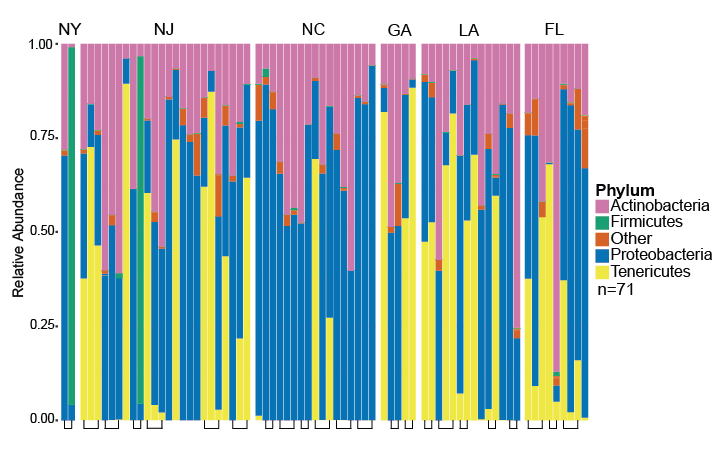

Supplement: FIG S3 [file msphere.00989-21-sf003.tif]

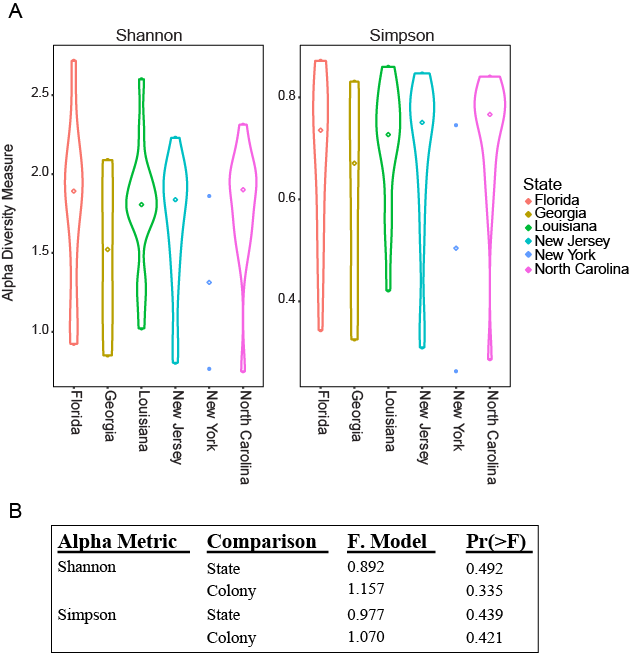

Supplement: FIG S4 [file msphere.00989-21-sf004.tif]

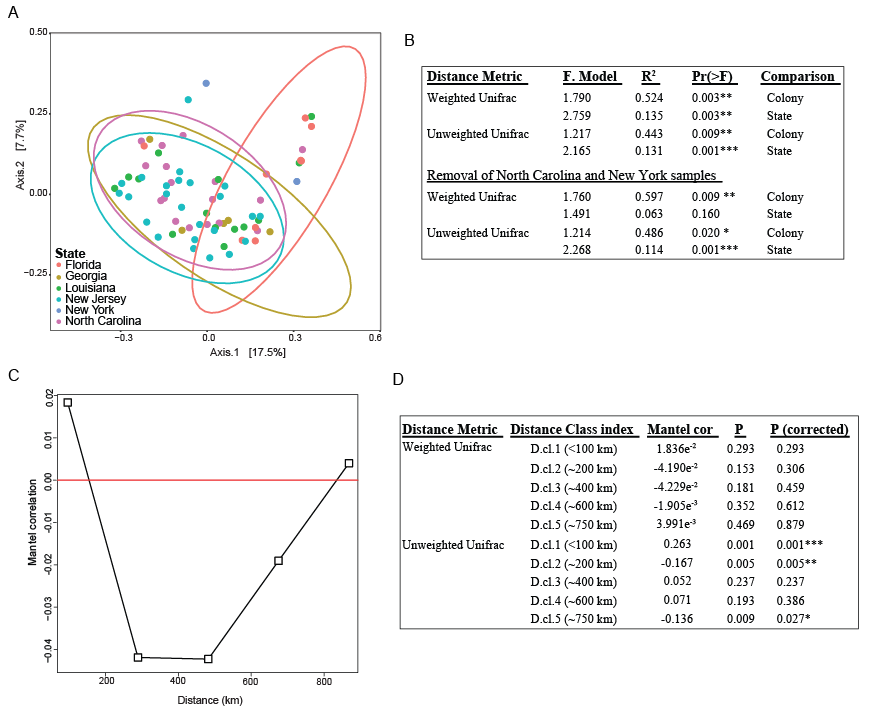

Supplement: FIG S5 [file msphere.00989-21-sf005.tif]

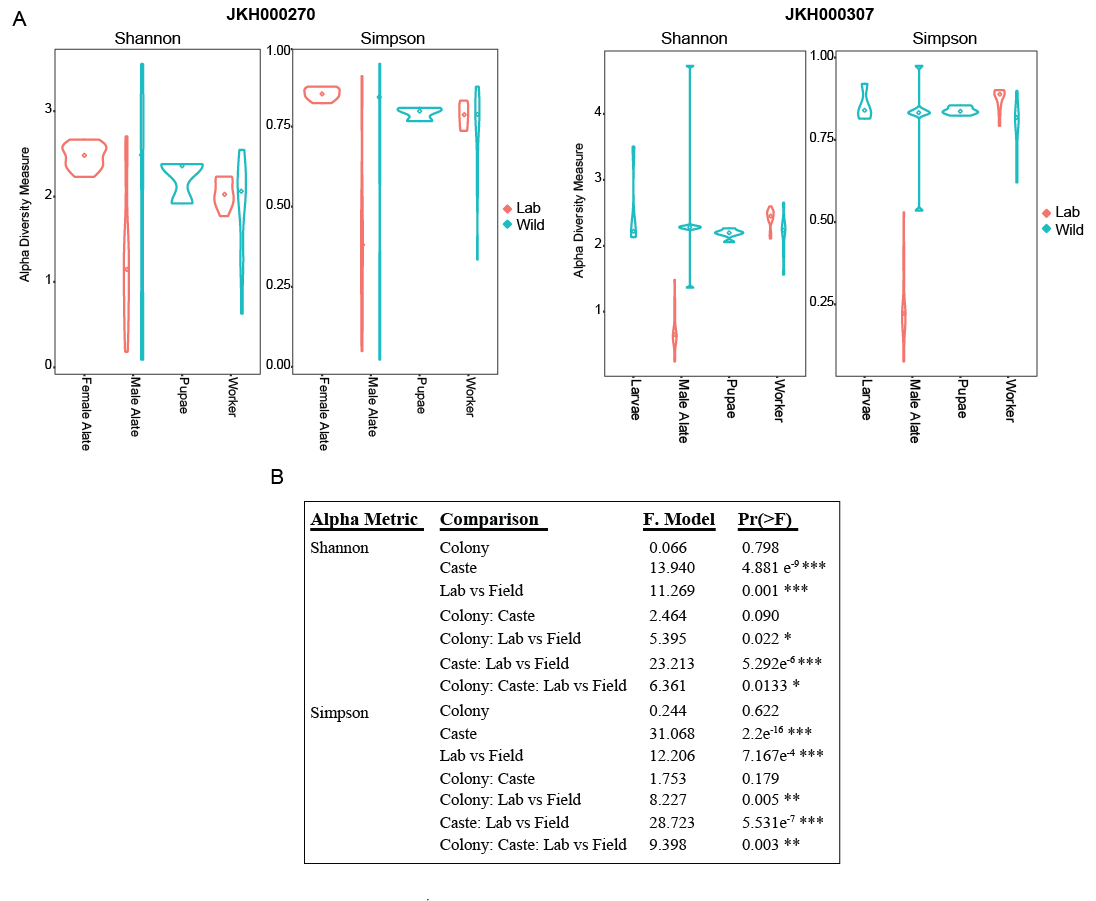

Supplement: FIG S6 [file msphere.00989-21-sf006.tif]

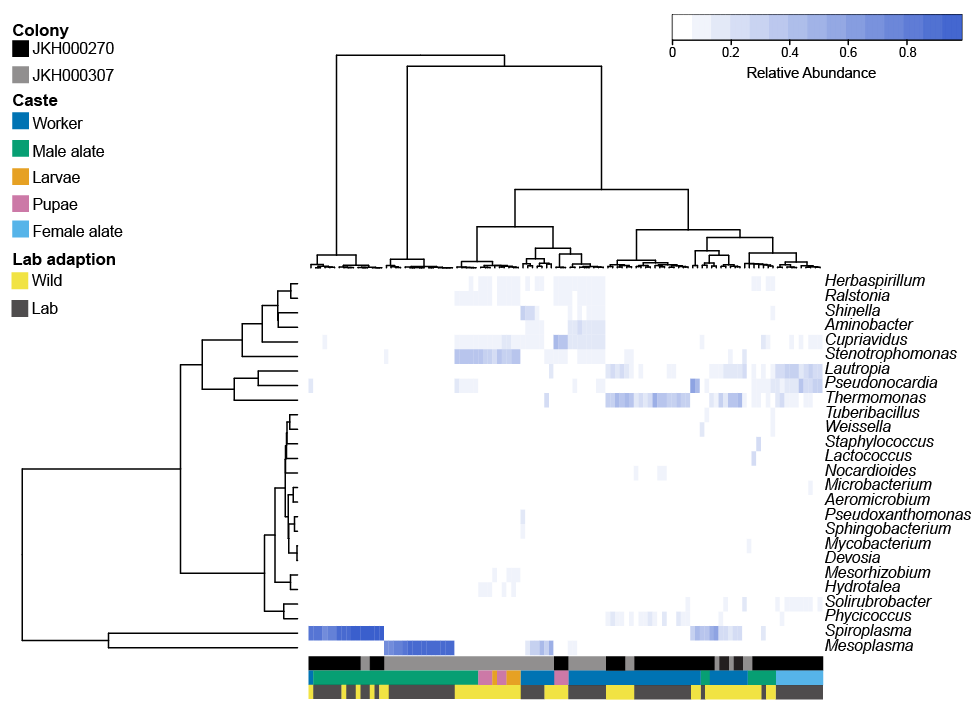

Supplement: FIG S7 [file msphere.00989-21-sf007.tif]

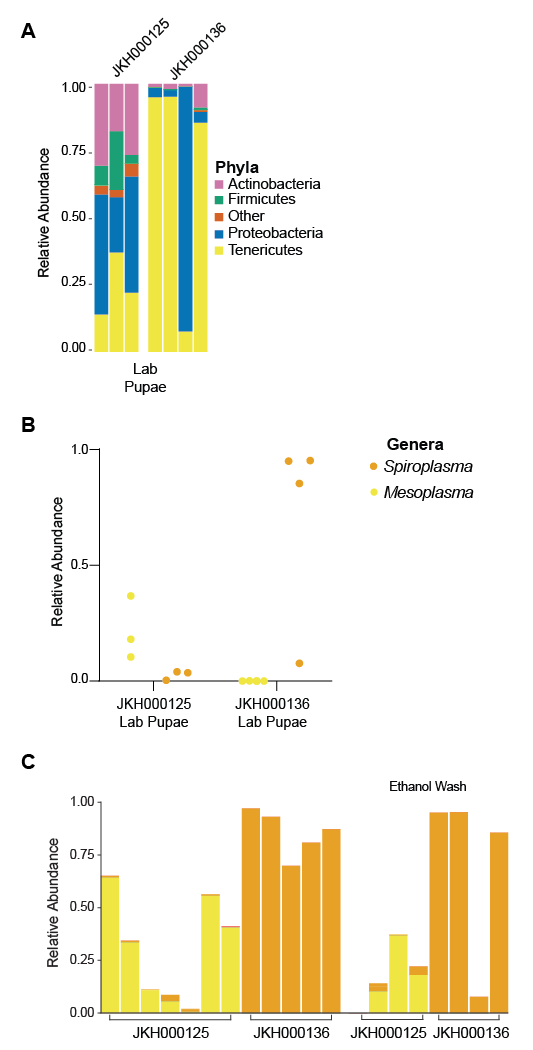

Supplement: FIG S8 [file msphere.00989-21-sf008.tif]

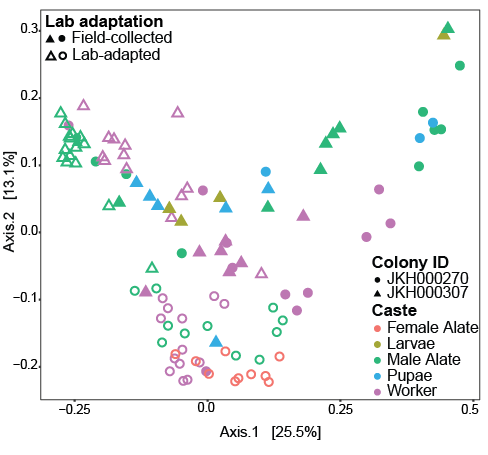

Supplement: FIG S9 [file msphere.00989-21-sf009.tif]

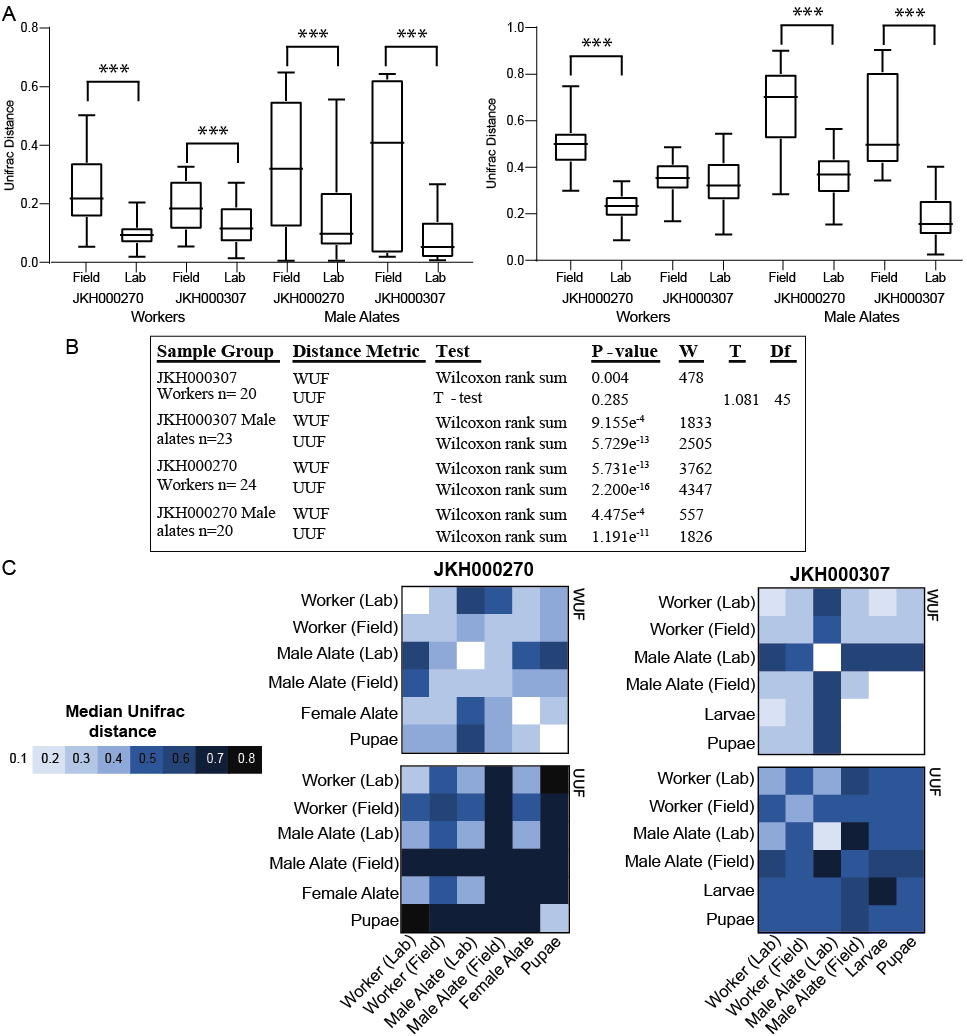

Supplement: FIG S10 [file msphere.00989-21-sf010.tif]

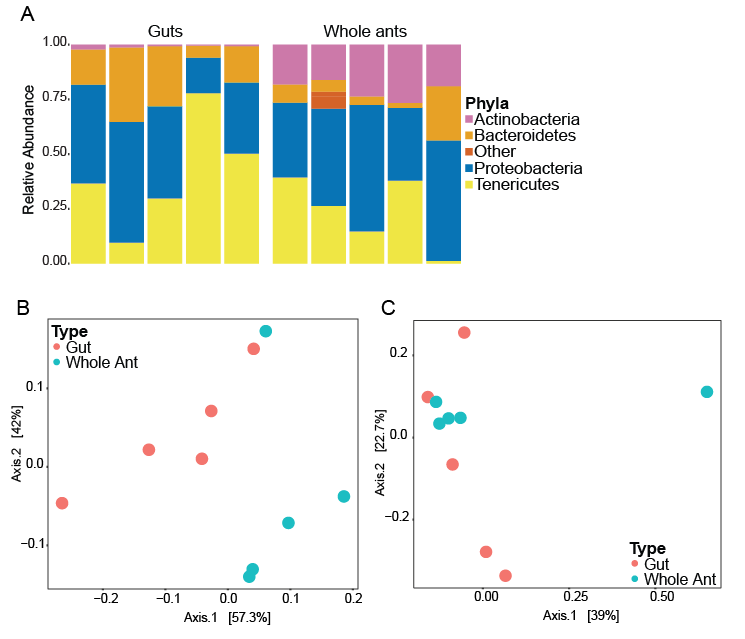

Supplement: FIG S2 [file msphere.00989-21-sf002.tif]
